# Spatial heterogenicity of tuberculosis and dengue in Nepal

**DOI:** 10.1038/s41598-025-24798-8

**Published:** 2025-11-20

**Authors:** Roshan Kumar Mahato, Kittipong Sornlorm, Kyaw Min Htike, Alex Bagas Koro, Rajitra Nawawonganun, Vijay Sharma, Alok Kafle

**Affiliations:** 1 Faculty of Public Health, Khon Kaen University, Khon Kaen, Thailand; 2 Kathmandu University School of Medical sciences, Dhulikhel, Nepal; 3 Department of Tropical Medicine, Faculty of Medicine, Khon Kaen University, Khon Kaen, Thailand

**Keywords:** SaTScan™, TB, Dengue, Nepal, Diseases, Risk factors

## Abstract

Nepal, a geographically diverse country in South Asia, faces significant public health challenges, including high burdens of tuberculosis (TB) and dengue. Understanding the spatial and temporal distribution of these issues are essential for developing effective interventions. This study aimed to identify spatial-temporal tuberculosis clusters for fiscal years (FY) 2020–2021 to 2022–2023 and dengue for 2021 to 2023 in Nepal using spatial-temporal analysis. A retrospective analysis using Kulldorff’s spatial-temporal scan statistics was conducted, offering a reference for infectious disease research and guiding policymakers in resource prioritization and targeted interventions. In addition, this study employed a discrete Poisson probability model to analyze the TB cases from FY 2020–2021 to 2022–2023 and dengue cases from 2020 to 2023 with a spatial window covering up to 50% of the population at risk. This study revealed significant geographic disparities in tuberculosis and dengue incidence across Nepal. For TB, primary clusters like Rautahat, Bara, Sarlahi, Mahottari and Kathmandu showed high Log-Likelihood Ratios (LLRs), indicating a persistent excess of observed cases, particularly in urban areas in FY 2022–2023. Secondary clusters also demonstrated elevated LLRs suggesting widespread TB risk. Similarly, dengue incidence was notably higher than expected in primary clusters such as Sankhuwasabha, Lalitpur, Bhaktapur and Kathmandu over three years, with urban areas experiencing sharp increases in 2022. This present study identified significant high-risk clusters for TB and dengue, emphasizing the need for targeted public health interventions in Nepal. Ongoing spatial-temporal analysis is crucial for adapting responses to evolving health challenges.

Nepal, a geographically diverse country in South Asia, faces significant public health challenges due to its varied climate, altitudinal gradients and population clusters concentrated in specific regions. These densely populated centres within large areas facilitate easier disease transmission, adding complexity to surveillance and control efforts^1^. Among the most urgent public health concerns are the substantial burdens of infectious diseases, including TB and dengue infections^2^. In 2022, Nepal reported 29,000 TB cases and over 40,000 dengue cases nationally highlighting the urgency of spatially targeted interventions^3^. These challenges place significant strain on the healthcare system of Nepal and adversely affect the overall well-being of the population^4^.

TB remains a major public health concern in Nepal, with its prevalence strongly influenced by environmental and socio-economic factors^5^. Despite global efforts to reduce TB incidence, certain regions, particularly in low-income and densely populated areas, continue to report high disease prevalence^6^. Identifying clusters of TB cases allows healthcare services to be targeted to the most affected regions, allowing prevention and treatment efforts where they are most urgently needed^7^.

Similarly, dengue fever, a mosquito-borne disease, has emerged as a significant health threat in Nepal^8^. The incidence of dengue has been closely linked to climatic conditions such as temperature and rainfall, as well as urbanization patterns^9–11^. In recent years, the country has seen periodic outbreaks of dengue, particularly in urban and semi-urban areas^12^. As the climate continues to change and urbanization accelerates, understanding the spatial-temporal patterns of dengue outbreaks becomes crucial for preventing further escalation^13^.

Both TB and dengue place significant burdens on Nepal’s healthcare system, with TB requiring long-term management and dengue necessitating rapid outbreak responses. These diseases serve as complementary case studies for spatial-temporal analysis, with TB reflecting socio-economic influences and dengue highlighting climate sensitivity. Their inclusion demonstrates the flexibility and applicability of the proposed methodology for addressing diverse drivers of disease distribution.

Our study provides an established methodological prototype for studying the spatial and temporal distribution of health concerns, offering a framework applicable to infectious disease surveillance. Given the significance of these health issues, spatial-temporal analysis provides a valuable tool for identifying clusters of TB and dengue^14^. Using Geographic Information Systems (GIS) and spatial scan statistics such as Kulldorff’s SaTScan has proven effective in detecting significant clusters of diseases^15^^16^. By applying these methods, it is possible to understand how health risks vary across time and space, allowing public health officials to tailor interventions accordingly. Therefore, this study addressed the lack of comprehensive spatial-temporal cluster analysis of TB and dengue in Nepal. This integrated approach can aid in formulating comprehensive public health policies to address infectious diseases with both acute and chronic impacts.

## Materials and methods

### Study area

Nepal, located between India and China’s Tibet Autonomous Region, spans 147,181 square kilometers, extending 885 km from east to west and 193 km from north to south. Nepal’s population is approximately 29 million (2021 Census) excluding about 2.2 million residing abroad^17^. Geographically, Nepal is situated between latitudes 26°22′ N to 30°27′ N, and longitudes 80°4′ E to 88°12′ E. Administratively, it is divided into 7 provinces and 77 districts, facilitating governance and resource management across its diverse landscapes.

### Source of data

**Fig. 1:**
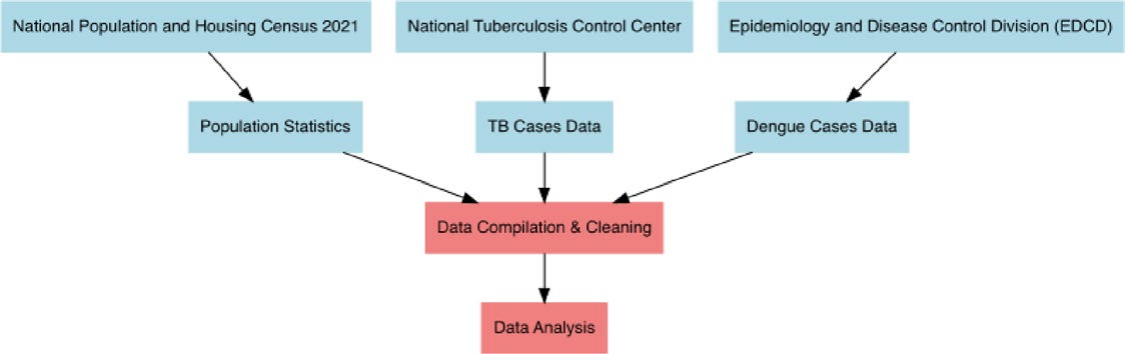
Data sources and flow for secondary data collection.

All secondary data for the study were collected from multiple sources. Population statistics were obtained from the National Population and Housing Census 2021^18^. Information on TB cases for the years 2021 to 2023 was sourced from the National Tuberculosis Control Center, Nepal^19^. Dengue data were provided by the Epidemiology and Disease Control Division (EDCD) under the Ministry of Health and Population (MOHP), Nepal^20^. The selected time period corresponds to the most recent and nationally representative datasets. It also aligns with intensified national response efforts for TB and dengue, allowing for timely analysis of evolving epidemiological patterns and spatial heterogeneity relevant to current public health priorities (Fig. 1).

### Data analysis

Kulldorff’s spatial-temporal scan statistics were used to identify statistically significant retrospective clusters of TB for FY 2020–2021 and FY 2022–2023, as dengue cases for 2020 and 2023 in Nepal. The analysis was conducted using SaTScan™ version 10.2.1, applying a discrete Poisson probability model, which assumes that the number of TB and Dengue cases in each district follows a Poisson distribution^21^. All 77 districts were included in the analysis to ensure full national coverage. Spatial clusters were identified based on case counts and population distribution, without prior selection criteria, to reduce bias and reflect natural disease clustering patterns.

Shape files were generated for each year using district populations and geographical coordinates (longitude and latitude) throughout the study period. A 50% maximum spatial and temporal window was selected based on SaTScan guidelines to detect both localized and large clusters. Sensitivity analyses using 30% and 40% thresholds produced consistent spatial patterns. The time unit was annual, reflecting the structure of the available case and population data. The log-likelihood ratio and relative risk (RR) were computed, and a standard Monte Carlo simulation with 999 repetitions was performed to test the null hypothesis of cases being randomly distributed. Clusters were considered statistically significant if the p-value was less than 0.05.

### Ethics approval

Ethical approval exemption has been granted by the Centre for Ethics in Human Research at Khon Kaen University, Thailand, with reference number HE 672,162.

## Results

### Descriptive summary of annual reported TB and dengue cases in Nepal (2020–2023)

**Fig. 2:**
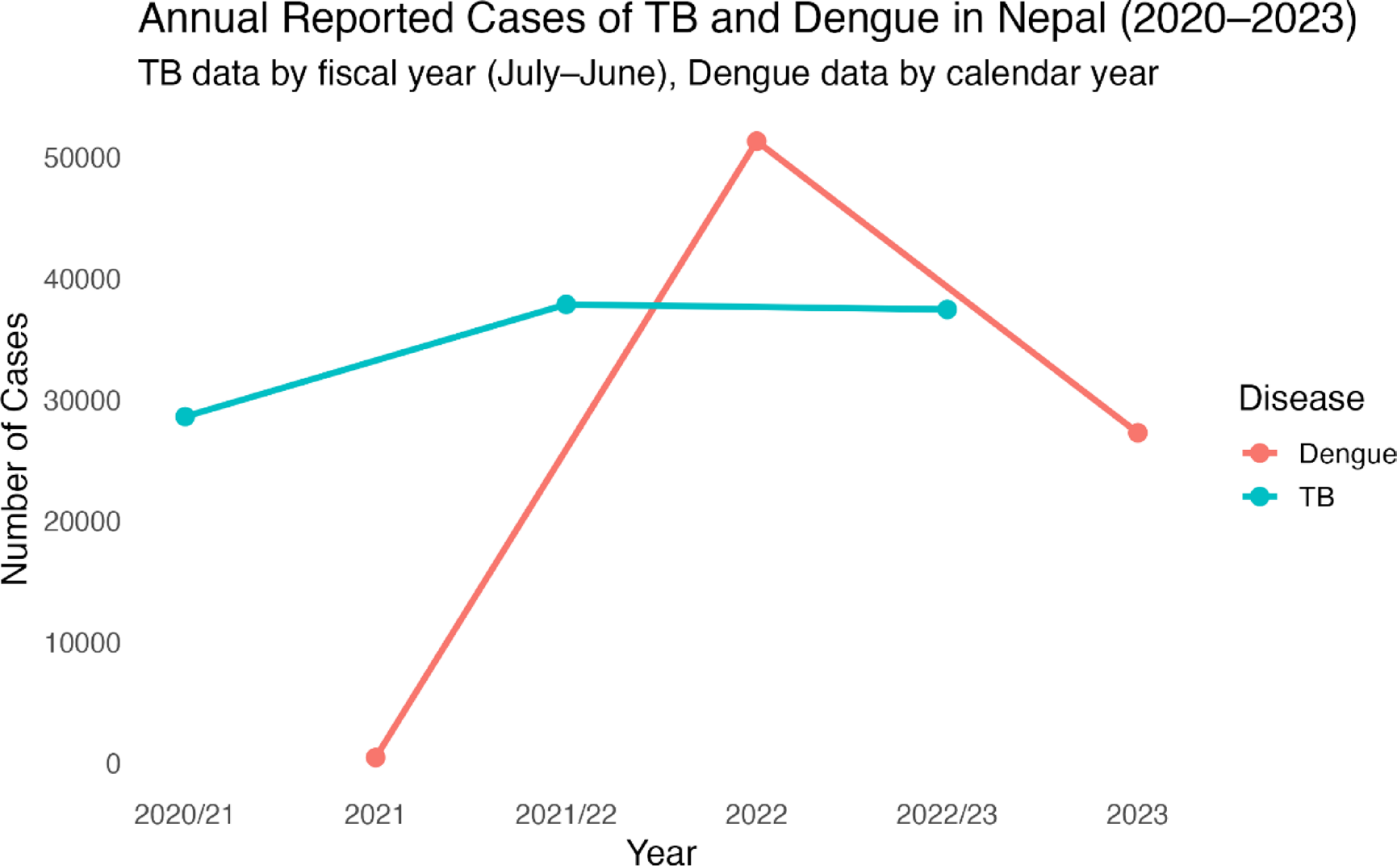
Annual reported cases of TB and Dengue in Nepal (2020–2023).

The reported cases of TB and Dengue in Nepal between 2020 and 2023 show distinct patterns. TB cases increased from 28,605 in the FY 2020–2021 to 37,861 in FY 2021–2022, then slightly decreased to 37,447 in FY 2022–2023 indicating a relatively stable but high TB burden. In contrast, Dengue cases were much lower in 2021, with 475 cases followed by a sharp rise to 51,331 cases in 2022, before declining to 27,270 in 2023. The TB data is reported by fiscal year (July-June) whereas Dengue data is reported by calendar year and the timeline in the plot aligns these for comparison (Fig. 2).

### Spatial-temporal scan analysis of TB clusters in FY 2020–2021, FY2021-2022 and FY 2022–2023

The results from the spatial analysis for TB prevalence in Nepal between FY 2020–2021 and FY 2022–2023 show significant differences across various clusters, with the LLR being an important indicator to assess the disparity between observed and expected TB cases. In FY 2020–2021, the primary cluster covering regions like Rautahat, Bara, Sarlahi, Mahottari, Makawanpur, Parsa, Lalitpur, Kabhrepalanchok, Sindhuli, Dhanusha, Bhaktapur and Kathmandu, the LLR was 308.42, which was significant. This indicated that the observed TB cases in this area were substantially higher than what was expected based on population size and other demographic factors. The LLR here showed a marked deviation from the expected TB cases suggesting a notably higher risk for TB in these regions. Among the secondary clusters, Kathmandu had an LLR of 225.79 with p-value < 0.001 indicating that the TB prevalence in the urban area was much higher than expected. Other secondary clusters such as Mahottari, Dhanusha, Sarlahi, Banke, Salyan, Bardiya and Dang also displayed notable positive LLRs, indicating higher-than-expected TB cases, suggesting that these areas should be further studied for potential risk factors.

In FY 2021–2022, the primary cluster continued to show a high TB prevalence with an LLR of 497.70 reflecting a significant increase in observed TB cases compared to the expected cases. This again emphasized the higher-than-expected TB burden in these regions highlighting them as critical areas for intervention. Secondary clusters such as Makawanpur, Lalitpur, Kathmandu, Banke, Salyan, Bardiya and Dang maintained elevated LLRs, indicating a continued excess of TB cases in these areas. Other clusters like Dhanusha, Mahottari and Sarlahi also showed significant but somewhat reduced LLRs compared to the first period, suggesting some improvement but still a notable burden of TB in these areas.

**Fig. 3:**
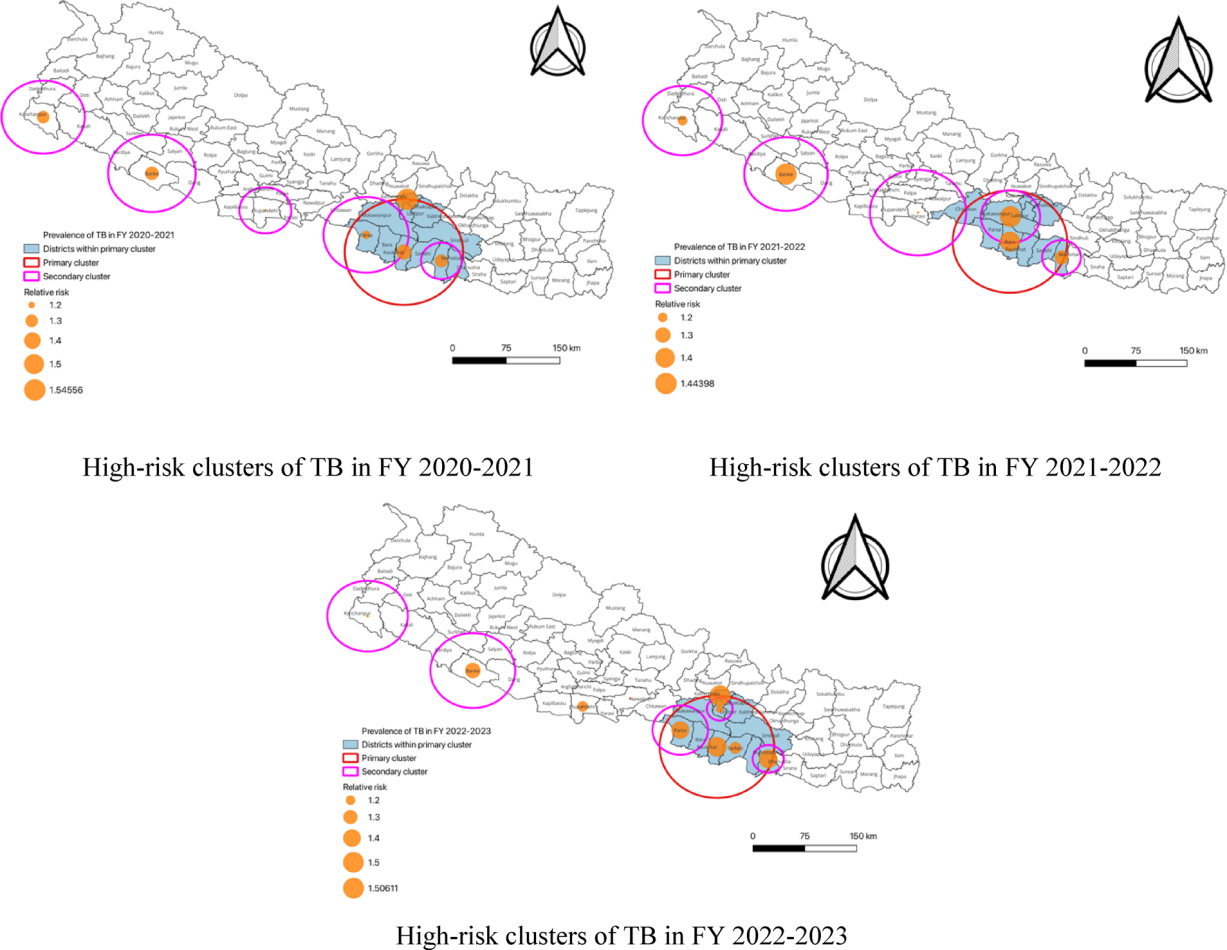
The space-time clusters of TB cases at the district level in Nepal, FY 2020–2021, FY 2021–2022 and FY 2022-2023^21,22^.

In FY 2022–2023, the primary cluster continued to show a high LLR of 624.48, indicating that the TB burden in these regions remained substantial. This LLR suggested a significant excess of observed TB cases compared to what was expected signalling persistent risk factors in the primary cluster. In secondary clusters, Kathmandu again had the highest LLR at 257.80 reflecting an even higher TB burden than anticipated. Regions like Parsa, Bara, Makawanpur, Dhanusha and Mahottari also exhibited elevated LLRs, showing that these areas continued to experience a higher-than-expected TB burden. Other regions, such as Rupandehi, Sarlahi, Lalitpur, Bhaktapur, Kanchanpur, Dadeldhura and Kailali, also showed. However, their LLR values were comparatively lower, indicating a slight reduction in TB burdens relative to previous periods (Table 1; Fig. 3).

**Table 1:** Significant high-rate TB clusters in Nepal detected by SaTScan from FY 2020–2021, FY 2021–2022 and FY 2022–2023.

Cluster	Location	Radius/KM	Prevalence of TB/100,000	Observed cases	Expected cases	LLR	RR	*p*-value
**01/07/2020- 30/06/2021**								
Primary cluster	Rautahat Bara Sarlahi Mahottari Makawanpur Parsa Lalitpur Kabhrepalanchok Sindhuli Dhanusha Bhaktapur Kathmandu	80.88	120.4	10,621	8654.61	308.42	1.36	< 0.001
Secondary cluster	Kathmandu	0	146.1	2981	2002.42	225.79	1.55	< 0.001
Secondary cluster	Mahottari Dhanusha Sarlahi	27.89	125.1	3048	2390.45	91.44	1.31	< 0.001
Secondary cluster	Banke Salyan Bardiya Dang	59.29	127.2	2513	1938.68	83.96	1.32	< 0.001
Secondary cluster	Parsa Bara Makawanpur Chitawan Rautahat	58.04	117.2	4001	3351.55	67.64	1.23	< 0.001
Secondary cluster	Kanchanpur Dadeldhura Kailali	55.91	125.6	1956	1528.13	58.38	1.30	< 0.001
Secondary cluster	Rupandehi Parasi Palpa	35.30	114.0	1998	1720.20	22.75	1.17	< 0.001
**01/07/2021-30/06/2022**								
Primary cluster	Bara Rautahat Parsa Makawanpur Sarlahi Lalitpur Kabhrepalanchok Bhaktapur Kathmandu Mahottari Chitawan	82.18	163.9	13,715	10873.60	497.70	1.41	< 0.001
Secondary cluster	Makawanpur Lalitpur Kathmandu Parsa Bara Bhaktapur	42.45	173.7	8521	6372.87	402.08	1.43	< 0.001
Secondary cluster	Banke Salyan Bardiya Dang	59.29	182.1	3597	2565.99	199.10	1.44	< 0.001
Secondary cluster	Dhanusha Mahottari Sarlahi	27.89	161.2	3925	3163.95	93.37	1.27	< 0.001
Secondary cluster	Kanchanpur Dadeldhura Kailali	55.91	154.5	2406	2022.60	36.30	1.20	< 0.001
Secondary cluster	Parasi Palpa Rupandehi Nawalpur Syangja Chitawan	68.85	146.4	4541	4030.62	34.90	1.14	< 0.001
**01/07/2022-30/06/2023**								
Primary cluster	Rautahat Bara Sarlahi Mahottari Makawanpur Parsa Lalitpur Kabhrepalanchok Sindhuli Dhanusha Bhaktapur Kathmandu	80.88	164.9	14,544	11329.81	624.48	1.46	< 0.001
Secondary cluster	Kathmandu	0	186.9	3813	2621.38	257.80	1.51	< 0.001
Secondary cluster	Parsa Bara Makawanpur	39.07	175.8	3310	2418.63	158.57	1.40	< 0.001
Secondary cluster	Dhanusha Mahottari	21.94	179.1	2819	2021.95	148.79	1.43	< 0.001
Secondary cluster	Banke Salyan Bardiya Dang	59.29	168.0	3318	2537.94	117.98	1.34	< 0.001
Secondary cluster	Rupandehi	0	155.6	1745	1440.58	31.40	1.22	< 0.001
Secondary cluster	Sarlahi	0	158.8	1369	1107.40	29.66	1.25	< 0.001
Secondary cluster	Lalitpur Bhaktapur	18.13	149.4	1469	1263.19	16.51	1.17	< 0.001
Secondary cluster	Kanchanpur Dadeldhura Kailali	55.91	143.9	2240	2000.49	14.61	1.13	< 0.001

LLR = Log Likelihood Ratio;

RR = Relative Risk; *p* < 0.05 indicates statistical significance.

KM = Kilometer.

### Results of dengue cluster detection in 2021, 2022 and 2023

The SaTScan analysis identified several significant spatial clusters of dengue cases across three consecutive years (2021, 2022 and 2023) including both primary and secondary clusters with varying geographical coverage. In 2021, the primary cluster of Sankhuwasabha exhibited an exceptionally high incidence of dengue, with a reported 66 cases compared to an expected 2.57 yielding an LLR of 155.15 and an RR of 29.62 indicating an alarmingly higher-than-expected dengue incidence in this area. Myagdi, another secondary cluster also reported a considerable increase in dengue incidence, with 53 observed cases compared to 1.74 expected cases, leading to an LLR of 132.60 and RR of 34.10. Other secondary clusters such as Okhaldhunga, Ramechhap, Khotang and Sindhuli experienced notable excesses in dengue cases, with observed incidences far surpassing the expected showcasing moderate to significant increases in risk.

In 2022, the urban regions of Lalitpur, Bhaktapur, Kathmandu and Makawanpur experienced a sharp rise in dengue incidence, with a staggering 36,485 observed cases compared to 6,145.13 expected cases. This resulted in an LLR of 48,464.20 and an RR of 18.07 indicating a pronounced surge in dengue incidence in these areas. Secondary clusters such as Gorkha, Lamjung, Dhading, Nawalpur, Tanahu, Parasi and Chitawan also demonstrated significant increases in dengue incidence, with observed cases outpacing the expected by notable margins. Darchula, despite having a lower observed incidence, still reported a significant excess of cases with an LLR of 15.93 and RR of 1.39.

**Fig. 4:**
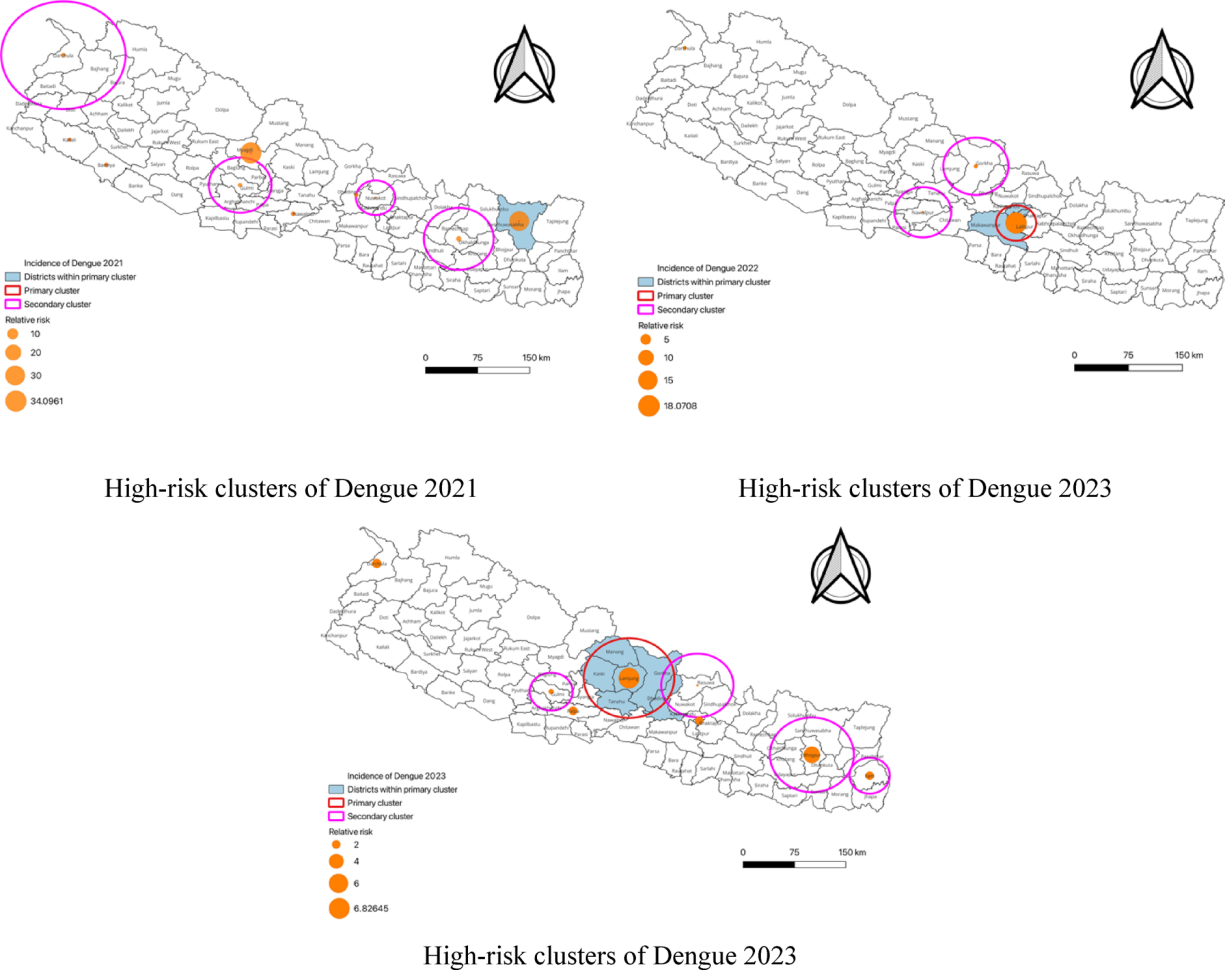
Spatial distribution of statistically significant Dengue clusters detected by purely spatial analysis in year 2021, 2022 and 2023^21,22^.

By 2023, the trend of high dengue incidence persisted with primary clusters such as Lamjung, Gorkha, Tanahu, Kaski, Manang and Dhading continuing to report high observed cases (7,955) compared to the expected incidence resulting in an LLR of 7,471.98 and RR of 6.83. Secondary clusters such as Bhojpur, Khotang, Dhankuta and Terhathum experienced a substantial increase in dengue incidence as well with an LLR of 4,795.60 and RR of 4.77 indicating heightened dengue risk in these areas. Other regions such as Bhaktapur, Palpa and Darchula also reported increases in dengue incidence with moderate to high RRs (1.33 to 2.18) pointing to consistent but more controlled risks (Table 2; Fig. 4).

**Table 2:** Significant Spatial clusters of dengue in Nepal based on maximum reported cluster size from 2021–2023.

Cluster	Location	Radius/KM	Incidence of Dengue/100,000	Observed cases	Expected cases	LLR	RR	*p*-value
**01/01/2021-31/12/2021**								
Primary cluster	Sankhuwasabha	0	41.8	66	2.57	155.15	29.62	< 0.001
Secondary cluster	Myagdi	0	49.6	53	1.74	132.60	34.10	< 0.001
Secondary cluster	Okhaldhunga Ramechhap Khotang Sindhuli	49.03	4.6	36	12.79	14.64	2.96	< 0.001
Secondary cluster	Gulmi Baglung Arghakhanchi Parbat Pyuthan Palpa	43.71	3.5	45	20.86	11.11	2.28	< 0.001
Secondary cluster	Darchula Bajhang Baitadi Dadeldhura	85.16	3.8	27	11.47	7.85	2.44	0.02
Secondary cluster	Kailali	0	3.4	31	14.73	7.08	2.18	0.037
**01/01/2022-31/12/2022**								
Primary cluster	Lalitpur Bhaktapur Kathmandu Makawanpur	27.11	1045.7	36,485	6145.13	48464.20	18.07	< 0.001
Secondary cluster	Gorkha Lamjung Dhading	43.76	245.5	1797	1289.39	91.49	1.41	< 0.001
Secondary cluster	Nawalpur Tanahu Parasi Chitawan	38.39	200.7	3623	3178.57	31.77	1.15	< 0.001
Secondary cluster	Darchula	0	244.7	326	234.63	15.93	1.39	< 0.001
**01/01/2023-31/12/2023**								
Primary cluster	Lamjung Gorkha Tanahu Kaski Manang Dhading	63.90	478.4	7955	1551.65	7471.98	6.83	< 0.001
Secondary cluster	Bhojpur Khotang Dhankuta Terhathum Sankhuwasabha Udayapur Sunsari	59.90	353.6	7080	1868.46	4795.60	4.77	< 0.001
Secondary cluster	Ilam, Panchthar	28.82	180.0	815	422.58	145.77	1.96	< 0.001
Secondary cluster	Bhaktapur	0	177.8	770	404.06	133.08	1.93	< 0.001
Secondary cluster	Palpa	0	185.7	456	229.11	87.93	2.01	< 0.001
Secondary cluster	Darchula	0	202.1	270	124.65	63.73	2.18	< 0.001
Secondary cluster	Gulmi Baglung Arghakhanchi	30.39	123.1	830	629.09	29.89	1.33	< 0.001
Secondary cluster	Rasuwa Nuwakot Sindhupalchok Kathmandu	51.02	102.5	2684	2444.46	12.53	1.11	< 0.001

LLR = Log Likelihood Ratio;

RR = Relative Risk; *p* < 0.05 indicates statistical significance.

KM = Kilometer.

## Discussion

This study identified significant spatial heterogeneity of TB and dengue in Nepal from FY 2020–2021 to FY 2022–2023 for TB and from 2021 to 2023 for dengue. These findings revealed substantial geographic disparities in disease burden, emphasizing the need for region-specific public health interventions. Understanding such clustering is especially critical in Nepal’s diverse topography where healthcare access is often limited in mountainous and hilly areas. In these regions, geographical barriers and limited infrastructure contribute to delayed diagnosis and treatment. Insights from this study can help design targeted interventions to improve healthcare access and disease control in both urban and remote settings. Our findings are consistent with studies conducted in countries such as Brazil^23^, Pakistan^24^^25^, Indonesia^26^^27^ and Thailand^28^ which also reported TB and dengue clusters in high-density urban areas using spatial scan statistics. Urban dengue clusters in Nepal resemble those reported in Thailand^29^ and the Philippines^30^ where urban heat islands, waste management gaps and rapid urbanization contribute to mosquito proliferation.

In our TB analysis, major urban centers like Kathmandu consistently emerged as primary high-risk clusters across all three years. This is likely due to a combination of rapid urbanization, high population density, internal migration and variability in healthcare access. Kathmandu hosts a large number of internal migrants particularly from rural areas, often living in crowded rental housing or informal settlements with limited ventilation where the conditions conducive to TB transmission. Secondary clusters such as Banke, Salyan, and Dang also exhibited persistently high relative risks suggesting that peri-urban and semi-rural areas remain epidemiologically vulnerable. These regions may serve as transit zones or economic hubs, increasing human mobility and contact networks which are known drivers of TB. Studies in India and Pakistan have similarly shown TB clusters forming along transport corridors and rapidly expanding urban fringes, where slum development, poverty and limited health infrastructure converge^24^^25^. In contrast, rural districts such as Kanchanpur and Dadeldhura showed moderate but concerning relative risks, potentially due to weaker disease surveillance, delayed diagnosis and limited access to TB care services. This observation aligns with findings from Ethiopia and Indonesia where rural TB hotspots were associated with geographic inaccessibility and health worker shortages^26^^31^. Moreover, rural patients may face longer travel times to diagnostic centers and lower awareness of TB symptoms contributing to diagnostic delays and ongoing transmission.

For dengue, clusters in the Kathmandu Valley (Kathmandu, Bhaktapur, Lalitpur) and surrounding districts were likely driven by urban infrastructure challenges such as poor drainage, stagnant water and inadequate waste management. These environmental conditions provide ideal breeding habitats for Aedes aegypti and Aedes albopictus mosquitoes, the primary vectors for dengue transmission. Additionally, high human population density, increased mobility and unregulated urban growth further accelerate disease transmission in these urban settings. A study from Dhaka, Bangladesh demonstrated a strong correlation between solid waste accumulation and increased vector density, contributing to high dengue incidence in slum neighborhoods^13^. The pattern observed in Kathmandu Valley is also consistent with findings from Thailand and Indonesia, where urban heat islands, irregular water storage practices, and intermittent water supply systems create conducive conditions for year-round dengue transmission^11^^32^. Moreover, climate variability such as increased rainfall and warmer temperatures is known to extend the breeding season and expand mosquito habitat ranges.

In contrast, rural clusters in Sankhuwasabha, Gorkha, and Lamjung may be driven by different but equally critical factors. These areas often have limited access to formal vector control programs, low health literacy, and infrequent health surveillance, leading to under-preparedness during outbreaks. In addition, changing climate patterns in mid-hill and mountainous regions including increased rainfall intensity and shifting temperatures are expanding the elevation range suitable for mosquito survival. A study reported that dengue incidence in Nepal has increasingly been observed in higher altitude districts, likely due to climate-induced ecological shifts^10^. Similar to our rural findings, research in Vietnam and India also indicated dengue outbreaks in previously non-endemic highland areas, attributed to climate change, forest encroachment and poor vector control infrastructure^33^^34^. These observations highlight the urgent need to adapt dengue control strategies in both urban and rural settings to account for environmental, socioeconomic and climatic vulnerabilities.

Climate change is an increasingly important driver of both TB and dengue transmission. Warmer temperatures, shifting rainfall patterns and extreme weather events extend mosquito breeding seasons and affect respiratory health, particularly in vulnerable populations. Climate-related migration and socioeconomic disruption can also increase TB risk especially in marginalized groups. Public health strategies must therefore account for environmental stressors in disease prevention planning. Although our study did not directly assess drug-resistant TB, multidrug-resistant (MDR) and extensively drug-resistant (XDR) forms remain a serious threat in Nepal. According to the National Tuberculosis Program, MDR-TB accounts for a significant share of new and retreatment cases. These strains pose additional challenges for TB control and underscore the importance of surveillance and early detection, particularly in high-risk clusters identified in this study. The variation in risk across clusters reinforces that a one-size-fits-all approach is inadequate. Urban areas may benefit more from infrastructure improvements, health system strengthening and public awareness campaigns. In contrast, rural areas require tailored interventions focused on mobile health services, vector surveillance and community engagement. Strengthening local health systems and empowering health workers are essential for early detection and sustained control.

## Conclusions

This study highlights persistent and emerging hotspots of TB and dengue in Nepal, particularly in urban and semi-urban districts. Spatial scan statistics revealed complex patterns of risk, shaped by urbanization, infrastructure, climate, and healthcare access. Limitations include the relatively short study period and the absence of individual-level covariates such as age, sex or comorbidities. However, the findings remain valuable for guiding future research and resource allocation. We recommend enhancing surveillance systems in high-risk areas, integrating climate resilience into disease control programs, and improving community-based interventions such as vector management and active TB screening. This spatially informed approach will help target resources more effectively and reduce the burden of infectious diseases across Nepal’s diverse geographic regions.

## Data Availability

All the data and codes are available in Zenodo (https://doi.org/10.5281/zenodo.14668782).
